# Neuronal CRMP2 phosphorylation inhibition by the flavonoid, naringenin, contributes to the reversal of spinal sensitization and arthritic pain improvement

**DOI:** 10.1186/s13075-022-02975-8

**Published:** 2022-12-23

**Authors:** Yue-Peng Jiang, Song Wang, Wei-Dong Lai, Xue-Qing Wu, Yan Jin, Zheng-Hao Xu, Aubin Moutal, Rajesh Khanna, Ki Duk Park, Zhi-Ming Shan, Cheng-Ping Wen, Jie Yu

**Affiliations:** 1 College of Basic Medical Science, College of Pharmaceutical Science, Key Laboratory of Neuropharmacology and Translational Medicine of Zhejiang Province, Zhejiang Chinese Medical University, Hangzhou, 310058 China; 2 Department of Pharmacology and Physiology, Saint Louis University - School of Medicine, Saint Louis, MO 63104 USA; 3 Department of Molecular Pathobiology, College of Dentistry, and NYU Pain Research Center, New York University, New York, 10010 USA; 4 Korea Institute of Science and Technology, Seoul, South Korea; 5 Department of Anesthesiology, Shenzhen People’s Hospital (The First Affiliated Hospital, Southern University of Science and Technology, The Second Clinical Medical College, Jinan University), Shenzhen, 518020 China

**Keywords:** Rheumatoid arthritis, Chronic pain, Central sensitization, CRMP2, Naringenin

## Abstract

**Background:**

Rheumatoid arthritis patients usually suffer from arthritic chronic pain. However, due to an incomplete understanding of the mechanisms underlying autoimmune disorders, the management of arthritic pain is unsatisfactory. Here, we investigated the analgesic effect and underlying mechanism of the natural flavonoid naringenin (NAR) in collagen-induced arthritis (CIA) pain.

**Methods:**

NAR was injected (i.p.) once per day for 42 days after initial immunization, and rats were sacrificed on the 28th (the 21st day after final immunization, PID 21) and 42nd days (PID 35). The inflammatory factors, central sensitization indicators, and CRMP2 phosphorylation, as well as the anti-rheumatoid activity and analgesic effect of NAR, were further investigated.

**Results:**

We found that NAR decreased the arthritis score and paw swelling, as well as the mechanical and thermal pain. The immunofluorescence results also showed a dose dependent effect of NAR on reducing the expressions of spinal cFos, IBA-1, and GFAP on the 28th (PID 21) and 42nd day (PID 35). NAR decreased the phosphorylation of CRMP2 S522 and the expression of the kinase CDK5 in the spinal dorsal horn, but pCRMP2 Y479 was unchanged. In addition, CRMP2 was co-localized with NEUN, but not IBA-1 or GFAP, indicating the involvement of neural CRMP2 phosphorylation in CIA-related pain. Finally, CRMP2 S522 phosphorylation selective inhibitor (*S*)-lacosamide also alleviated arthritic pain.

**Conclusions:**

Taken together, our results demonstrate that NAR alleviates inflammation and chronic pain in CIA model, which might be related to its inhibition of neuronal CRMP2 S522 phosphorylation, potentially mitigating the central sensitization. Our study provide evidence for the potential use of NAR as non-opioid-dependent analgesia in arthritic pain.

**Supplementary Information:**

The online version contains supplementary material available at 10.1186/s13075-022-02975-8.

## Background

Rheumatoid arthritis (RA) is a chronic autoimmune disease with an incidence ranging from 0.3 to 4.2% in different populations [1, 2]. The most prominent characteristics of RA are symmetrical arthritic pain and swelling (polyarthritis) in the joints of hands, wrists, ankles, and knees. RA patients usually show unilateral arthritis chronic pain and oligoarthritis (fewer than five joints, during the first 6 months), which impairs body function and quality of life [3]. Current clinical treatments for RA patients mainly aim to control inflammation in an effort to reduce or alleviate disease progression. Medications include traditional anti-rheumatic drugs (e.g., methotrexate, hydroxychloroquine and sulfasalamide), biological agents (e.g., etanercept and adalimumab), targeted anti-rheumatic drugs (e.g., tofacitinib), and non-steroidal anti-inflammatory drugs, as well as hormones [4]. Although these medications reduce arthritic pain, not all arthritic individuals report pain relief. Thus, persistent arthritic pain remains a major concern that affects patients’ lives, their daily activities, and their physical and mental health [5]. As one of the most common complaints of RA patients, arthritic chronic pain afflicts 97% with early RA and is the primary reason for seeking medical advice in clinic [6]. Randomized clinical trials showed that early use of disease-modifying anti-rheumatic drugs (DMARDs) and glucocorticoids could improve pain outcomes, but the analgesic effects of the latter lasts less than 3 months [7]. A cross-sectional survey conducted in the United States in 2018 showed that up to 74% RA patients are dissatisfied with their medication due to an aggravation of their chronic pain [8]. Moreover, 12–20% of patients that suffered prolonged pain of differing intensities usually have no signs of inflammation, which suggests that RA pain might not only be attributed to peripheral inflammation but also may be due to changes in the central nervous system (CNS) [9–11].

Central sensitization, descending facilitation, and inhibition constitute the three main categories of endogenous modulation of pain in the CNS [12, 13]. Spinal glial activation and subsequent secretory activities play a leading role in central sensitization [14], while peripheral mechanisms of arthritic chronic pain include direct inflammatory sensitization of joint nociceptors [15, 16]. It was reported that electroacupuncture regulated the activation of microglia cells in the spinal cord of rats with spinal nerve ligation, thereby affecting the release of inflammatory factors and inhibition of pain-related signal transduction pathways [17, 18]. Reports also indicate that 41% of patients with RA exhibit central sensitization [19]. In addition, neuro-psychological and immune factors are also involved in RA pain chronification [20]. However, the limited effectiveness of arthritis chronic pain management is due to an incomplete understanding of pain mechanisms underlying pathological progression in RA. Moreover, whether modulation of central sensitization can alleviate chronic pain in RA still remains elusive.

Collapsin response mediator protein (CRMPs) 2 is a widely expressed phosphoprotein in the nervous system [21]. Our previous studies have thus far confirmed that the regulation of CRMP2 phosphorylation in DRG sensory neurons is an intrinsic pathological event participating in the establishment of chronic neuropathic pain.

Uncoupling CRMP2 from the CaV2.2 channel complex in DRG neurons suppressed inflammatory and neuropathic pain [22, 23]. We also reported that phosphorylated CRMP2 directly regulates presynaptic excitatory neurotransmission in the spinal cord, exhibiting a significant effect on the central regulation of pathological pain [24, 25]. These suggested that CRMP2 could be a potential therapeutic target for arthritic pain. Naringenin (NAR) is a dihydroflavonoid found in grapefruit, tomatoes, grapes, and citrus rutaceae. It has a variety of pharmacological activities including antibacterial, analgesic, or immunomodulatory [26, 27]. NAR can cross the blood-brain barrier and exert different neuronal effects through its ability to interact with the protein kinase C signaling pathway [28]. We previously also reported that NAR can provide analgesia by inhibiting NaV1.8 voltage-gated sodium channel in DRG sensory neurons [29]. Moreover, NAR specifically binds CRMP2 to reduce its phosphorylation [30], which might be hopeful to regulate central sensitization and deliver safe and non-opioid-dependent analgesia in RA chronic pain.

In this study, we investigated the analgesic effect of NAR on chronic pain-like behaviors in collagen-induced RA rats by mitigating central sensitization. Our results highlight novel approaches for the pain management of autoimmune diseases. Here, we demonstrated that (i) the central sensitization of rheumatoid arthritis was closely related to arthritis chronic pain, (ii) NAR suppressed spinal central sensitization in collagen-induced RA chronic pain, and (iii) the inhibition of RA central sensitization by NAR was mainly manifested by its inhibition of spinal neuronal CRMP2 phosphorylation.

## Methods

### Animals

Female Wistar rats (aged 6–8 weeks, 180 ± 15g) and male DBA/1 mice (aged 6–8 weeks, 20 ± 2g) were purchased from Shanghai SLAC Laboratory Animal Co., Ltd., (Shanghai, People’s Republic of China) and housed in an animal facility maintained at 25°C (humidity, 50–60%) under a 12-h light-dark cycle with free access to food and water. All experiments involving animals were approved and performed in accordance with the institutional guidelines for animal care of the animal ethics committee of Zhejiang Chinese Medical University (20190909-07) and international guidelines using animals (ARRIVE 2.0), to minimize the number and suffering of animals used in experiments.

### Experimental design and treatment strategies

**Fig. 1:**
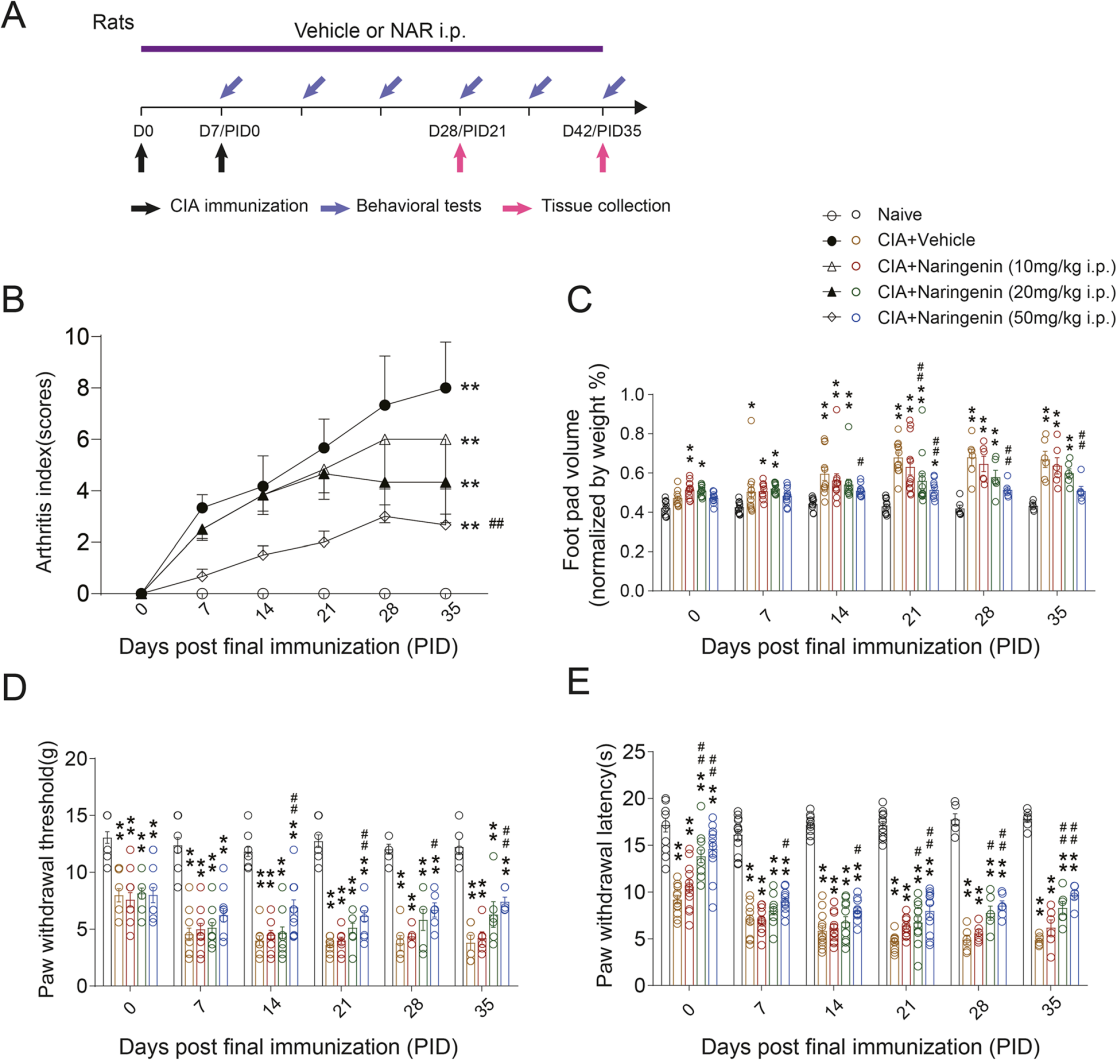
Collagen treatment successfully induced RA, and NAR treatment alleviated the degree of arthritic score and pain in CIA rats. **A** Experimental design and treatment strategies. **B** Arthritic score in CIA rats. **C** CIA rats toe volume. **D** Mechanical pain threshold in CIA rats. **E** Thermal withdrawal latency in CIA rats. *P < 0.05 vs. naive group, **P < 0.01 vs. naive group. ^#^*P* < 0.05 vs. CIA model group, ^##^*P* < 0.01 vs. CIA model group, by repeated-measures two-way ANOVA followed by post hoc Dunnett’s multiple comparisons test (Rats from PID0 to PID21 are *n* = 12/group. From PID28 to PID35 are *n* = 6/group). PID: days post the second immunization. In this and subsequent figures, the experimenters were blinded to the treatment condition(s)

Rats were randomly divided into five groups by random number table method: naive (*n* = 12), collagen-induced RA group (*n* = 12), NAR (10mg/kg)-treated collagen-induced RA group (*n* = 12), NAR (20mg/kg)-treated collagen-induced RA group (*n* = 12), and NAR (50 mg/kg)-treated collagen-induced RA group (*n* = 12). Both non-NAR treated (naive and CIA model groups) and NAR treated animals were handled in the same way. Rats in the naive and CIA-induced model groups were intraperitoneally injected with vehicle (10% Tween 80+10%DMSO+80% normal saline), and rats in NAR groups were intraperitoneal injected with NAR (10% Tween 80+10%DMSO+80% normal saline+10, 20 and 50 mg/kg NAR, respectively) daily from day 1 to day 42 after initial immunization. The injections were made in the morning (8:00–12:00) on the same days which behavioral tests occurred in the afternoon (13:00–18:00). On the 28th (PID 21) and 42nd (PID 35) day following the induction of RA, the rats were sacrificed (Fig. 1A). Mice with the successful induction of RA were randomly divided into two groups: collagen-induced RA group (*n* = 8), (S)-LCM (20 mg/kg, diluted with normal saline)-treated collagen-induced RA group (*n* = 8). Mice in the (S)-LCM group were intraperitoneally injected with (S)-LCM daily from day 1 to day 42. On the 42nd (PID 21) day following the initial induction of RA, the mice were sacrificed, as shown in Fig. 7A.

### Reagents and antibodies

Immunization Grade Bovine Type II Collagen (Cat. No. 20021; Chondrex), Naringenin (Cat. No. B21596; Shanghai Yuanye Bio-Technology Co., Ltd), and (S)-lacosamide ((*S*)-LCM) (provided by the laboratory of Dr. Ki Duk Park, Korea Institute of Science and Technology, Seoul, South Korea) were used in this study. Western blot experiments were performed using the following antibodies: rabbit anti-CRMP2 polyclonal antibody (1:5000; Cat. No. C2993; Sigma), rabbit anti-CRMP2 (Ser-522) phospho-specific polyclonal antibody (1:1000; Cat. No. CP2191; ECM), rabbit anti-CRMP2 (Tyr-479) phospho-specific polyclonal antibody (1:2000; Jia Xuan biological), rabbit anti-CDK5 monoclonal antibody (1:2000; Cat. No. ab207238; Abcam), and rabbit anti-GAPDH monoclonal antibody (1:1000; Cat. No. 5174; CST). Immunofluorescence experiments were performed using the following antibodies: rabbit anti-IBA1 antibody (1:200; Cat. No. 019-19741; Wako Chemicals), goat anti-GFAP antibody (1:200; Cat. No. ab53554; Abcam), rabbit anti-cFos antibody (1:200; Cat. No. ab208942; Abcam), rabbit anti-CRMP2, rabbit anti-pCRMP2 S522; goat anti-rabbit IgG H&L (Alexa Fluor® 488) (1:200; Cat. No. ab150077; Abcam); and donkey anti-goat IgG H&L (Alexa Fluor® 488) (1:200; Cat. No. ab150129; Abcam).

### Collagen-induced rheumatoid arthritis in rats and mice

In this study, we followed Brand and Miyoshi’s method to establish an animal model of collagen-induced arthritis, and female rats (more sensitive) were used to establish the RA model [31–33]. Briefly, bovine type II collagen was dissolved in 0.05 M acetic acid to a final concentration of 4 mg/mL and kept away from light overnight at 4 °C. Subsequently, the acetic acid solution of bovine type II collagen was emulsified in a 1:1 ratio with the Freund’s incomplete adjuvant, and the final concentration of the emulsion was 2 mg/ml. Accordingly, on day 0 (200μL) and day 7 (100μL), the emulsions were injected subcutaneously into the rat tail for immunization. Besides, the DBA/1 male mice is also a commonly used species in CIA model [34]. Accordingly, on day 0 (100 μL) and day 21 (100 μL), the emulsions were injected subcutaneously into the mice tail for immunization.

### Behavioral tests

#### Mechanical withdrawal threshold

From the day of initial immunization, the mechanical withdrawal threshold was measured every seven days, and the test time was arranged from 13:00 to 18:00. The experimenter was blind to the treatment groups. The rats or mice were adapted to the environment in the Plexiglas chamber for 15–30 min, until the end of their exploratory response in the cage, and then the pain threshold test was conducted in silence. The pain threshold area is fixed in the center of the sole, avoiding the insensitive pad. Rats with eight different strengths of von Frey hairs test (rat (g): 0.4, 0.6, 1.0, 2.0, 4.0, 6.0, 8.0, 15.0; mouse (g): 0.04, 0.07, 0.16, 0.4, 0.6, 1.0, 1.4, 2.0). The rats were tested starting at 2.0 g (for rat) or 0.4 g (for mouse) at intervals of more than 5 min. If the animal appears to shrink, lick, shake, or escape violently in a circle, a von Frey filament with reduced force was applied. If a negative response was elicited, a filament with increased force was applied. The improved up and down method was used to determine pain threshold [35]. Rats from PID0 to PID21 are *n* = 12/group. From PID28 to PID35 are *n* = 6/group.

#### Thermal withdrawal latency test

From the day of initial immunization, thermal withdrawal latency test was performed in the afternoon every 7 days (13:00 to 18:00). The rats or mice were adapted to the environment in the Plexiglas chamber for 15–30 min, until the end of their exploratory response in the cage, and then the thermal pain threshold test was conducted in silence. The rats were tested using a hot plate and placed on a hot metal plate with a temperature of 50.0 ± 0.5 for observation. Paw withdraw latency (PWL) is defined as the time to start jumping, retraction, or licking a paw. In order to avoid tissue damage caused by thermal stimulation, the test time should not exceed 30 s. The testers blind to the treatment groups. Each animal was repeated three times and the mean was taken as the thermal withdrawal latency. Behavioral tests were performed 3 h after the administration of the intervention in each group. Rats from PID0 to PID21 are *n* = 12/group. From PID28 to PID35 are *n* = 6/group.

### Arthritic scoring

For the severity score of CIA rats’ posterior paw arthritis, the three experimental researchers evaluated the score and calculated the mean arthritic score [36]. Severity was scored on a scale of 0–4 points: 0 = no redness or swelling; 1 = slight swelling of ankle or redness of foot; 2 = progressive swelling, inflammation, and redness from the ankle to the middle of the soles of the feet; 3 = the whole foot is swollen and inflamed; and 4 = swelling and inflammation of the entire foot, with a loss of mobility. The arthritic index was calculated as a total score of 16 points/animal (4 points per limb). Successful model induction was verified when the arthritis score was greater than 6 scores. Rats from PID0 to PID21 are *n* = 12/group. From PID28 to PID35 are *n* = 6/group.

### Paw edema measurement

On the day before modeling, the left and right hind paws of each rat were marked, and the bilateral paw edema was measured with volume measuring instrument (YLS-7A), which was repeated three times and the average value was taken. Paw edema was then measured on days 7 (post the second/final immunization day 0, PID 0), 14 (PID 7), 21 (PID 14), 28 (PID 21), 35 (PID 28), and 42 (PID 35) after initial immunization using the same method. The mean value of each measurement for each animal was compared to its weight at the corresponding time point to remove the heterogeneity between the groups due to weight. Rats from PID0 to PID21 are *n* = 12/group. From PID28 to PID35 are *n* = 6/group.

### Sample preparation

To investigate the effect of NAR in different time points of the disease, we added an additional time point, 28 days (PID 21) after initial immunization in the CIA model, when the CIA-induced animals had the lowest values of mechanical and thermal pain thresholds, for immunohistochemistry and western blot experiments. Briefly, after 28 (PID 21) and 42 days (PID 35) of modeling, half of the rats were selected for biochemical analysis. Six rats from each group were executed at day 28 (PID 21), and the remaining six rats from each group were repeatedly measured for swelling and pain-related behavior longitudinally and then executed on day 42 (PID 35) after initial immunization. The rats were deeply anesthetized by isoflurane overdose. Cyclic citrullinated peptide (CCP) and anti-type II collagen antibody in serum were determined by ELISA. All mice in the (*S*)-lacosamide application experiment were sacrificed on day 42 (PID 21), and the spinal cord and spinal dorsal horn between L4 and L6 segments were carefully excised for immunofluorescence and western blot analysis, respectively.

### Immunofluorescence and laser confocal microscopy

Spinal cord segments between L4 and L6 were removed and fixed overnight in 4% paraformaldehyde. Dehydration with 25–30% sucrose the next day and wait for complete dehydration. The completely dehydrated spinal cord was prepared into 16 μm frozen sections for immunofluorescence staining. Digital pathological section (fluorescence) scanning analyzer (VS120-S6-W, OLYMPUS) was used. The expression of CRMP2 (1: 200, Cat. #C2993, Goat anti rabbit IgG, Alexa Fluor™ 647, #ab150079), pCRMP2 S522 (1: 200, Cat. #CP2191), cFos (1: 200, Cat. #ab208942), IBA1 (1: 200, Cat. #019-19741), and GFAP (1: 200, Cat. #ab53554) in the spinal dorsal horn were observed. The mean immunofluorescence intensity capture and analysis of microglial biomarker IBA1 and astrocyte biomarker GFAP, CRMP2, and pCRMP2 S522 were performed by the Image J software (National Institutes of Health, Bethesda, Maryland). Slice from L4 to L6 spinal cords of each animal of different groups were harvested, and 3 different fields of each slice in the spinal dorsal horn were randomly selected for semi-quantitative evaluation. The average fluorescence intensity value of the selected 3 different fields were taken and defined as one biological replicate (sample). All values were normalized to the average value of the samples from naive animals (*n* = 5). Quantification of the immunofluorescence data was carried out blindly with respect to treatments. The co-localization of CRMP2 and microglia, CRMP2 and astrocytes, and CRMP2 and neuron were observed by laser confocal microscope (LSM880, Zeiss).

### Western blot preparation and analysis

As described previously [25], spinal dorsal horn tissue lysates prepared from adult Wistar rats were generated by homogenization and sonication in RIPA buffer (Cat. #P0013B, Beyotime biotechnology). Protease inhibitors (Cat. #B14002; Bimake), phosphatase inhibitors (Cat. #B15002, Bimake), and pierce™ universal nuclease for cell lysis (Cat. #88700, Thermo Scientific) were used. The protein concentration was determined by BCA assay. Proteins were separated by sodium dodecyl sulfate polyacrylamide gel (12.5%) electrophoresis and then were transferred to nitrocellulose membranes by wet transfer at 100 v. Membranes were incubated in blocking solution (PBS, 0.1% Tween 20, 5% nonfat dried milk, 1 h) and then incubated (overnight, 4 °C) with specific antibodies: rabbit anti-CRMP2 polyclonal antibody (1:5000; Cat. #C2993; Sigma), rabbit anti-CRMP2 (Ser-522) phospho-specific polyclonal antibody (1:1000; Cat. #CP2191; ECM), rabbit anti-CRMP2 (Tyr-479) phospho-specific polyclonal antibody (1:2000; Jia Xuan biological), rabbit anti-CDK5 monoclonal antibody (1:2000; Cat. #ab207238; Abcam), and rabbit anti-GAPDH monoclonal antibody (1:1000; Cat. #5174; CST). After washing, samples were incubated (1 h, room temperature) with secondary antibodies: goat anti-rabbit IgG (H&L) (1:5000; Cat. #926-32211; LI-COR). After the film was scanned in Odyssey fluorescence imaging system, quantitative analysis was performed by the Image J software.

### Type II collagen antibodies and cyclic citrullinated peptide detection

Cyclic citrullinated peptide and anti-type II collagen antibody in serum were determined by ELISA. ELISA kit was used according to the instructions.

### Statistical analysis

All results were expressed as mean ± SEM. The behavioral tests among different groups were assessed by repeated measures two-way analysis of variance (ANOVA) with post hoc Dunnett’s multiple comparisons (time and group). For non-parametric data, the Kruskal-Wallis comparison or repeated measures Freidman test was used, followed by Dunn test for post hoc test. Besides, one-way ANOVA was used for assessment of other indicators among groups, followed by Dunnett’s test for post hoc test. In all calculations, a difference at *P* < 0.05 was regarded as significant. Statistical analysis was performed blindly on these independent values. The PASS software was used to determine the sample size (*n* > 5) to reach a significant level *α* = 0.05 and power (1-β) = 0.8. All data were plotted in GraphPad Prism 9 (GraphPad Software, San Diego, CA, USA).

## Results

### Naringenin reduced the arthritic score and alleviated pain-like behaviors in CIA rats

We first quantified the concentration of anti-type II collagen antibodies and cyclic citrullinated peptides in serum of rats with ELISA on the 42nd (PID 35) day after initial immunization. We found a significantly higher concentration of antibodies to type II collagen and CCP in the CIA model group compared to our sham group (Supplementary Fig. 1A and B). Next, we examined the effect of NAR on arthritic scores, plantar swelling, and pain-like behaviors of rats after CIA immunization. Rats progressively developed arthritis after the second injection of collagen (final immunization), evidenced by redness and swelling in their hind paws, and a restriction of their ability to move (Fig. 1B, Supplementary Fig. 2A and B). The arthritis scores of rats in the CIA+Vehicle group increased from day 14 (PID 7) to day 42 (PID 35) after initial immunization (primary immune response) (PID 7: 3.33 ± 0.51 vs. 0.00 ± 0.00; CIA+Vehicle group vs. Naive group; *P* < 0.01; *n* = 12) (Fig. 1B). In contrast, arthritic scores were significantly reduced in rats treated with 50 mg/kg NAR (PID 7: 0.67 ± 0.28 vs. 3.33 ± 0.51; CIA+50 mg/kg NAR group vs. CIA+Vehicle group; *P* < 0.01; *n* = 12). Fifty milligram/kilogram of NAR treatment also significantly reduced the paw edema from day 21 (PID 14) to day 42 (PID 35) (PID 14: 0.51 ± 0.00 vs. 0.60 ± 0.00; CIA+50 mg/kg NAR group vs. CIA+Vehicle group; *P* = 0.022; *n* = 12) (Fig. 1C), suggesting that the treatment reduced collagen-induced inflammation.

Pain is one of most disabling manifestations in patients with arthritis. CIA rats developed mechanical allodynia (PID 0: 7.98 ± 0.58g vs. 13.06 ± 0.51g; CIA+Vehicle group vs. Naive group; *P* < 0.01; *n* = 12) and thermal hyperalgesia (PID 0: 9.19 ± 0.46s vs. 17.15 ± 0.74s; CIA+Vehicle group vs. Naive group; *P* < 0.01; *n* = 12) from day 7 (PID 0) after the initial immunization and the pain thresholds kept being lower than baseline over time. Treatment with 50 mg/kg NAR significantly reduced mechanical allodynia from day 21 (PID 14) (until day 42 (PID 35)) (PID 14: 6.92 ± 0.66g vs. 3.99 ± 0.42g; CIA+50 mg/kg NAR group vs. CIA+Vehicle group; *P* < 0.01; *n* = 12), as well as thermal hyperalgesia from day 7 (PID 0) (until day 42 (PID 35)) after the initial immunization (PID 0: 14.59 ± 0.79s vs. 9.19 ± 0.46s; CIA+50 mg/kg NAR group vs. CIA+Vehicle group; *P* < 0.01; *n* = 12) (Fig. 1D, E). In rats treated with 20 mg/kg NAR, no effect was detected on mechanical allodynia but we observed a significant reduction of thermal hyperalgesia from day 28 (PID 21) to day 42 (PID 35) (Fig. 1D, E). These results showed that NAR might be a promising therapeutic to mitigate the pain-like symptoms of collagen-induced RA.

### Naringenin-attenuated spinal central sensitization in CIA rats

**Fig. 2:**
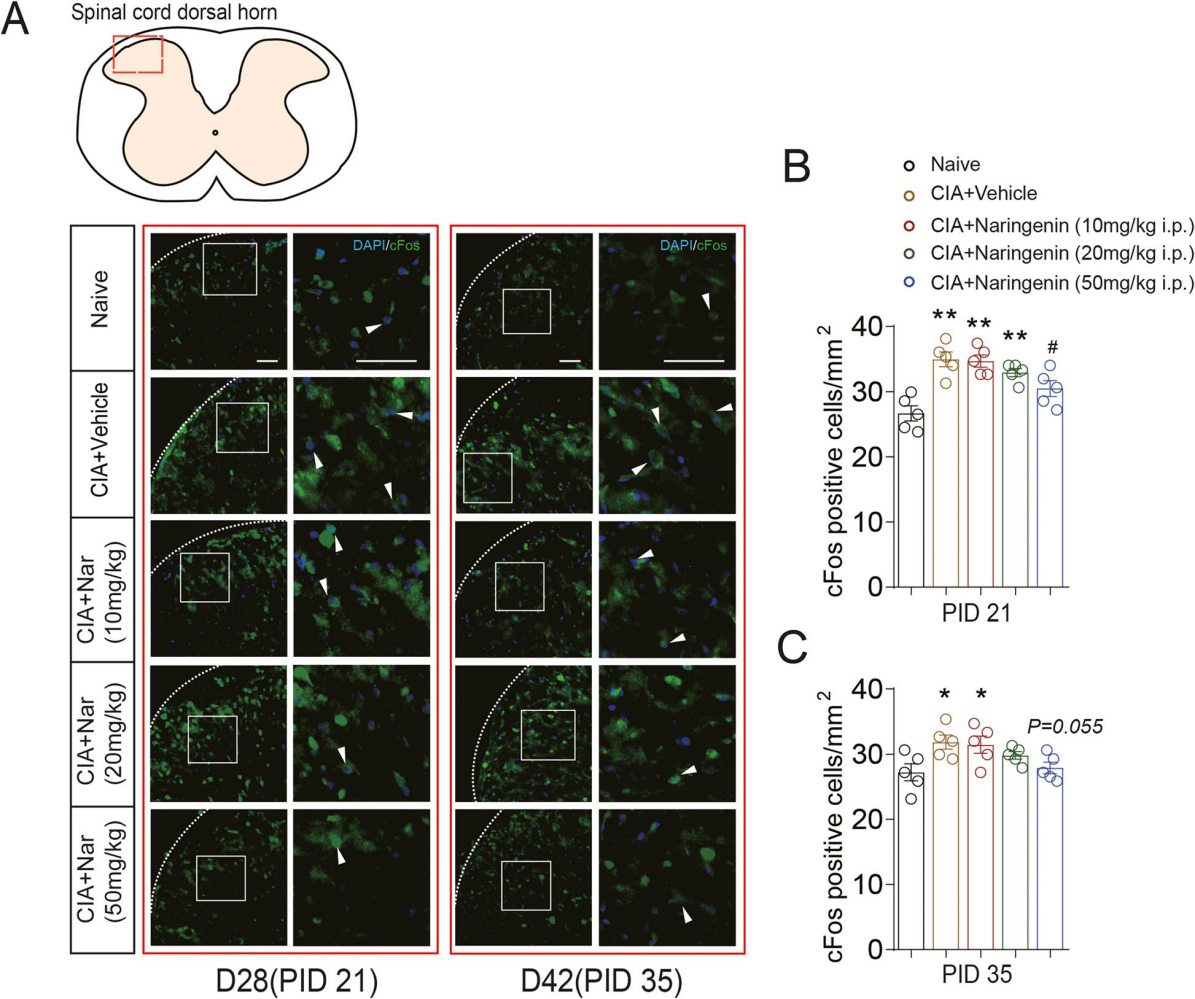
Naringenin decreased the expression of cFos in the spinal cord dorsal horn in CIA rats. **A** Representative micrographs of the expression of cFos in the spinal cord dorsal horn on days 28 (PID 21) and 42 (PID 35). **B**, **C** Expression of cFos in the spinal cord dorsal horn in CIA and NAR groups on the day 28 (PID 21) and day 42 (PID 35). *P < 0.05 vs. naive group, ** *P* < 0.01 vs. naive group, ^#^*P* < 0.05 vs. CIA model group, by repeated-measures one-way ANOVA followed by post hoc Dunnett’s multiple comparisons test. The dotted white lines lineate the edge of the section. The second column is a higher magnification of the white box indicated in the first column images. All scale bars are 50 μm. The relative mean fluorescence intensity of cFos were quantified using the Image J software. The data are presented as means ± SEM (*n* = 5 rats per group)

Persistent pain may lead to central sensitization. cFos protein is encoded by immediate early gene *cFos*, which expresses rapidly and transiently when neurons respond to stimuli and is used as a molecular marker for neuronal activation and central sensitization [37]. In our model of arthritis, results from immunofluorescence assay showed an increase of cFos expression in the dorsal spinal cord at days 28 (PID 21) and 42 (PID 35) after the initial immunization (PID 21: 34.97 ± 1.13 vs.26.67 ± 1.17; CIA+Vehicle group vs. Naive group; *P* < 0.01) (Fig. 2A, B) (PID 35: 31.84 ± 1.08 vs.27.21 ± 1.31; CIA+Vehicle group vs. Naive group; *P* = 0.021) (Fig. 2A, C). Fifty milligram/kilogram of NAR treatment reverted cFos expression with a significant reduction at day 28 (PID 21) and a significant downward trend at day 42 (PID 35) after the initial immunization (PID 21: 30.48 ± 1.21 vs. 34.97 ± 1.13; CIA+50 mg/kg NAR group vs. CIA+Vehicle group; *P* = 0.021) (Fig. 2A, B) (PID 35: 27.89 ± 0.89 vs. 31.84 ± 1.08; CIA+50 mg/kg NAR group vs. CIA+Vehicle group; *P* = 0.055) (Fig. 2A, C). Thus, NAR might alleviate central sensitization in the CIA rat model of arthritis.

**Fig. 3:**
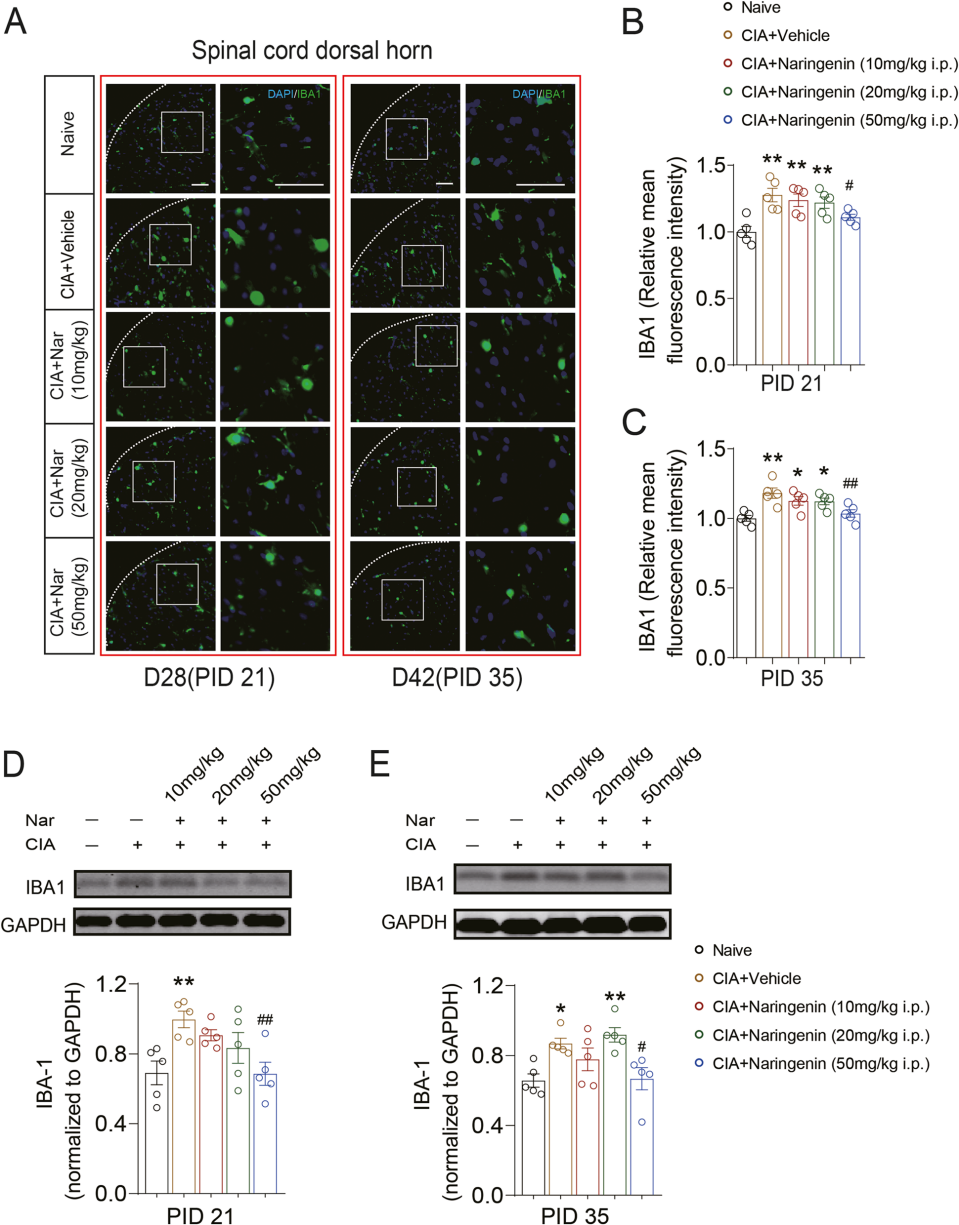
Naringenin decreased the expression of IBA-1 in the spinal cord dorsal horn in CIA rats. **A** The expression of IBA-1 in the spinal cord dorsal horn on days 28 (PID 21) and 42 (PID 35) were examined by immunofluorescence. **B**, **C** Expression of IBA-1 in the spinal cord dorsal horn in CIA and NAR groups on the day 28 (PID 21) and day 42 (PID 35). **D**, **E** Representative Western blots showing the expression of IBA-1 in the spinal cord dorsal horn in CIA and NAR groups on days 28 (PID 21) and 42 (PID 35). *P < 0.05, **P < 0.01 vs. naive group, ^#^*P* < 0.05, ^##^*P* < 0.01 vs. CIA model group, by repeated-measures one-way ANOVA followed by post hoc Dunnett’s multiple comparisons test. All scale bars are 50 μm. The relative mean fluorescence intensity of IBA-1 was quantified using the Image J software. The data are presented as means ± SEM (*n* = 5 rats per group)

IBA-1 and GFAP are markers for microglia and astrocytes, respectively, which are two cell populations that are activated in chronic pain and participate in central sensitization. Therefore, we next investigated the effect of NAR on the activation of glial cells in the spinal dorsal horn of CIA model rats. The expression of spinal IBA-1 in CIA rats increased on day 28 (PID 21) (1.28 ± 0.05 vs.1.00±0.04; CIA+Vehicle group vs. Naive group; *P* < 0.01) and day 42 (PID 35) (1.18 ± 0.04 vs. 1.00 ± 0.02; CIA+Vehicle group vs. Naive group; *P* < 0.01) after initial immunization, and 50 mg/kg NAR significantly attenuated the IBA-1 expression (PID 21: 1.11 ± 0.02 vs. 1.28 ± 0.05; CIA+50 mg/kg NAR group vs. CIA+Vehicle group; *P* = 0.036) (Fig. 3A, B, D) (PID 35: 1.04 ± 0.03 vs. 1.18 ± 0.04; CIA+50 mg/kg NAR group vs. CIA+Vehicle group; *P* < 0.01) (Fig. 3A, C, E).

**Fig. 4:**
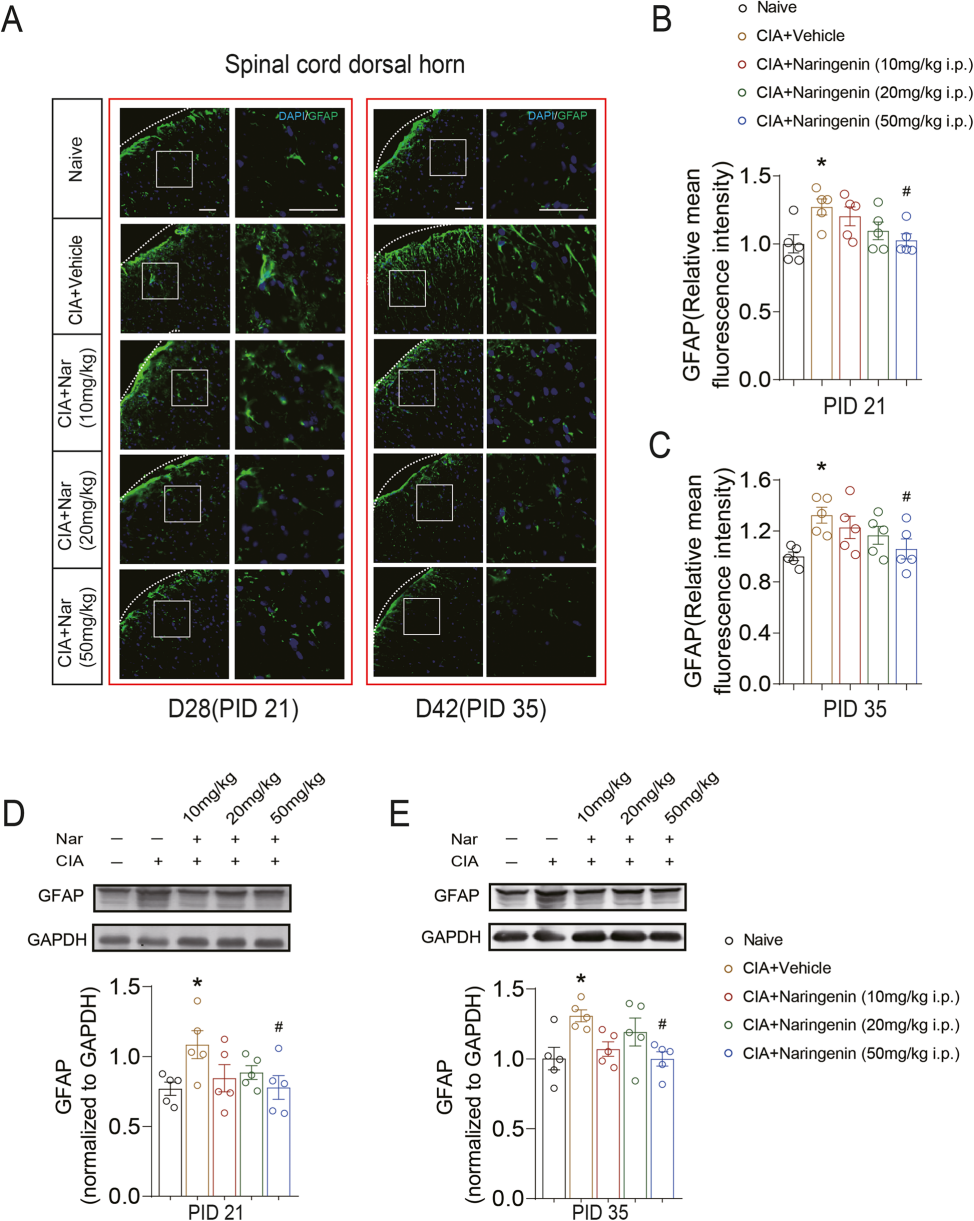
Naringenin decreased the expression of GFAP in the spinal cord dorsal horn in CIA rats. **A** The expression of GFAP in the spinal cord dorsal horn on the day 28 (PID 21) and day 42 (PID 35) were examined by immunofluorescence. **B**, **C** Representative Western blots showing expression of GFAP in the spinal cord dorsal horn in CIA and NAR groups on days 28 (PID 21) and 42 (PID 35). **D**, **E** Representative Western blots showing expression of GFAP in the spinal cord dorsal horn in CIA and NAR groups on the day 28 (PID 21) and day 42 (PID 35). *P < 0.05 vs. naive group, ** *P* < 0.01 vs. naive group, ^#^*P* < 0.05 vs. CIA model group, by repeated-measures one-way ANOVA followed by post hoc Dunnett’s multiple comparisons test. All scale bars are 50 μm. The relative mean fluorescence intensity of GFAP were quantified using the Image J software. The data are presented as means ± SEM (*n* = 5 rats per group)

In addition, spinal GFAP expression was also significantly increased in the CIA+Vehicle group rats on days 28 (PID 21) and 42 (PID 35) (PID 21: 1.27 ± 0.06 vs.1.00 ± 0.07; CIA+Vehicle group vs. Naive group; *P* = 0.022) (PID 35: 1.32 ± 0.06 vs.1.00 ± 0.03; CIA+Vehicle group vs. Naive group; *P* = 0.012), while 50 mg/kg NAR significantly attenuated the GFAP expression both on days 28 (PID 21) and 42 (PID 35) (PID 21: 1.03 ± 0.05 vs. 1.27 ± 0.06; CIA+50 mg/kg NAR group vs. CIA+Vehicle group; *P* = 0.044) (Fig. 4A, B, D) (PID 35: 1.06 ± 0.08 vs. 1.32 ± 0.06; CIA+50 mg/kg NAR group vs. CIA+Vehicle group; *P* = 0.045) (Fig. 4A, C, E). These results indicated that NAR could potentially inhibit central sensitization in CIA-induced arthritic pain.

### Naringenin reduced the phosphorylation of neuronal, but not microglial or astrocyte, CRMP2 in spinal cord of CIA rats

**Fig. 5:**
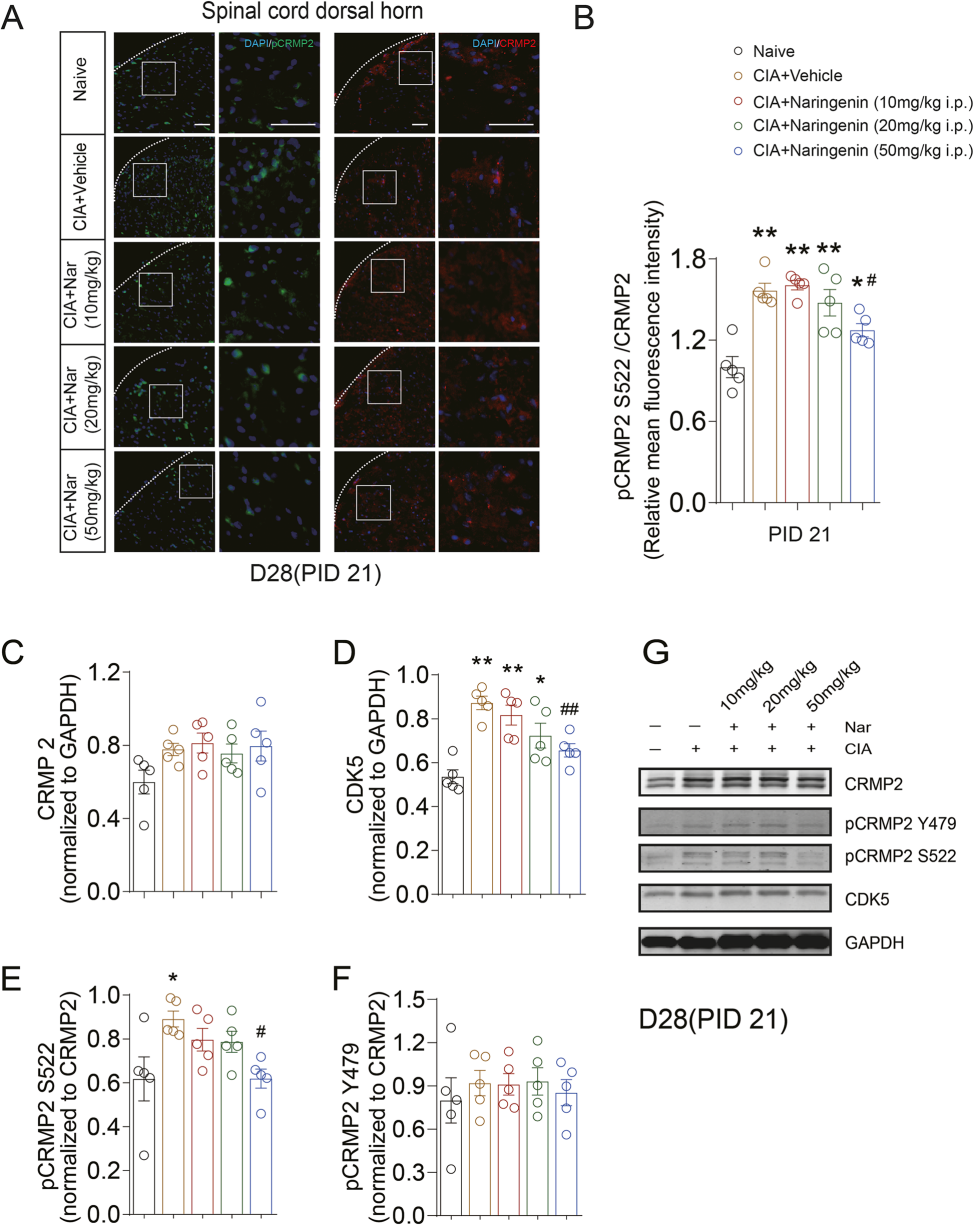
Naringenin reduces phosphorylation of CRMP2 S522 in spinal cord dorsal horn of CIA rats on day 28 (PID 21) following the first day of initial immunization. **A**, **B** The expression levels of CRMP2 and pCRMP2 S522 in the spinal cord dorsal horn on day 28 (PID 21) were examined by immunofluorescence. Representative Western blots showing expression of CRMP2 (**C**), CDK5 (**D**), pCRMP2 S522 (**E**), and pCRMP2 Y479 (**F**) in the spinal cord dorsal horn in CIA and NAR groups on day 28 (PID 21). **G** Representative blots are shown. *P < 0.05 vs. naive group, **P < 0.01 vs. naive group, ^#^*P* < 0.05 vs. CIA model group, ^##^*P* < 0.01 vs. CIA model group, by repeated-measures one-way ANOVA followed by post hoc Dunnett’s multiple comparisons test. The data are presented as means ± SEM (*n* = 5 rats per group). All scale bars are 50 μm. The relative mean fluorescence intensity of pCRMP2 S522 were quantified using the Image J software. The data are presented as means ± SEM (*n* = 5 rats per group)

**Fig. 6:**
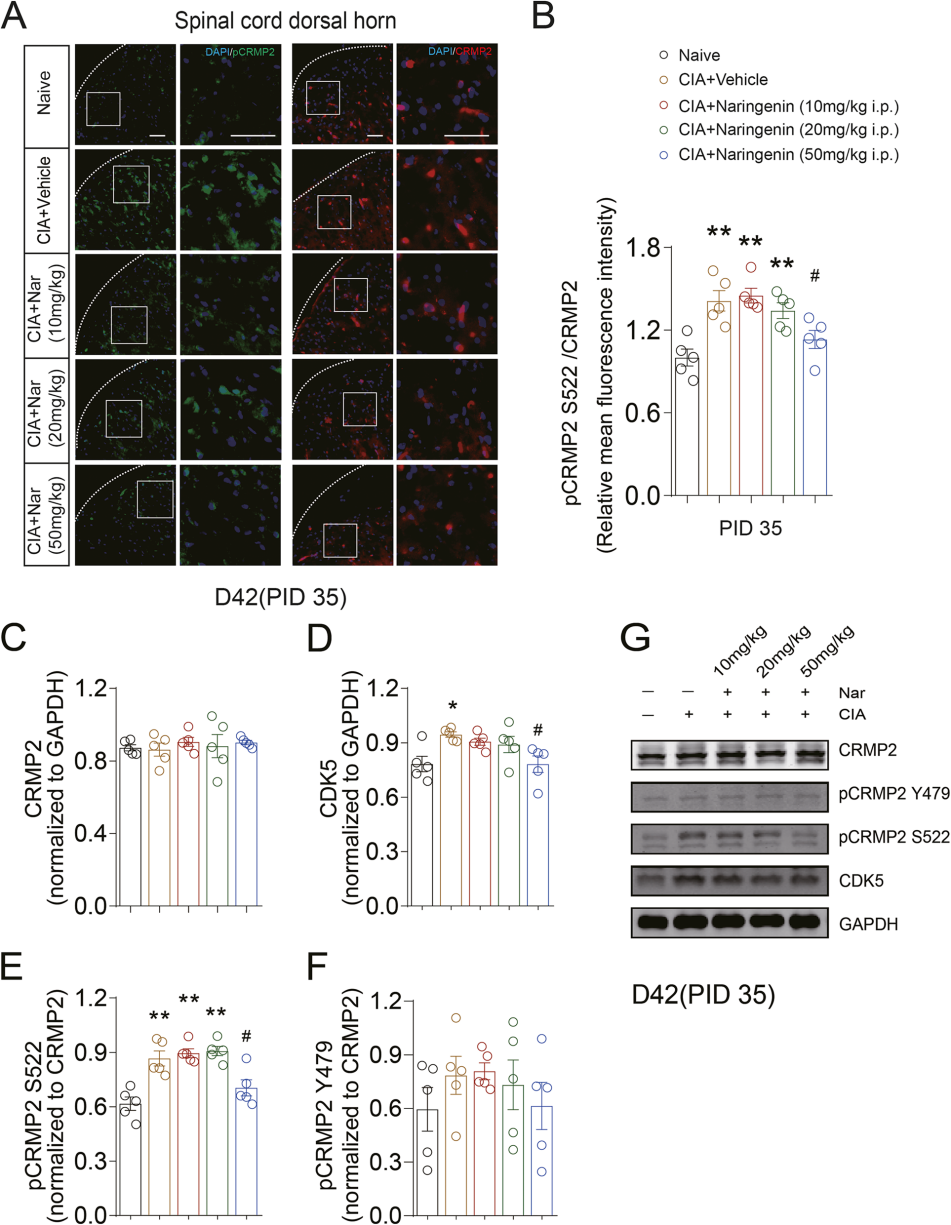
Naringenin reduces phosphorylation of CRMP2 in spinal cord dorsal horn of CIA rats on day 42 (PID 35) following the first day of initial immunization. **A**, **B** The expression levels of CRMP2 and pCRMP2 S522 in the spinal cord dorsal horn on day 42 (PID 35) were examined by immunofluorescence. Representative Western blots showing expression of CRMP2 (**C**), CDK5 (**D**), pCRMP2 S522 (**E**), and pCRMP2 Y479 (**F**) in the spinal cord dorsal horn in CIA and NAR groups on day 42 (PID 35). **G** Representative blots are shown. *P < 0.05 vs. naive group, **P < 0.01 vs. naive group, ^#^*P* < 0.05 vs. CIA model group, ^##^*P* < 0.01 vs. CIA model group, by repeated-measures one-way ANOVA followed by post hoc Dunnett’s multiple comparisons test. The data are presented as means ± SEM (*n* = 5 rats per group). All scale bars are 50 μm. The relative mean fluorescence intensity of pCRMP2 S522 were quantified using the Image J software. The data are presented as means ± SEM (*n* = 5 rats per group)

We further explored whether the analgesic effect of NAR in our model of RA chronic pain could also be given rise with CRMP2 phosphorylation as another marker of central sensitization. Consistent with our recently published findings [24], we did not find any CRMP2 expression in spinal microglia or astrocytes (Supplementary Fig. 3A and B). In addition, we found colocalization of CRMP2 with spinal dorsal horn neurons (Supplementary Fig. 3C). The immunofluorescence results showed the expression ratio of pCRMP2 S522 increased in the spinal dorsal horn on days 28 (PID 21) (1.57 ± 0.05 vs. 1.00 ± 0.08; CIA+Vehicle group vs. Naive group; *P* < 0.01) (Fig. 5A, B) and 42 (PID 35) (1.41 ± 0.07 vs. 1.00 ± 0.06; CIA+Vehicle group vs. Naive group; *P* < 0.01) (Fig. 6A, B) after initial immunization. In addition, the Western blot showed the expression of pCRMP2 S522 increased in the spinal dorsal horn on days 28 (PID 21) (0.89 ± 0.04 vs. 0.62 ± 0.10; CIA+Vehicle group vs. Naive group; *P* = 0.016) (Fig. 5C, E, G) and 42 (PID 35) (0.87 ± 0.04 vs. 0.62 ± 0.04; CIA+Vehicle group vs. Naive group; *P* < 0.01) (Fig. 6C, E, G) after initial immunization. The immunofluorescence results showed our treatment with 50 mg/kg NAR significantly decreased the expression ratio of pCRMP2 S522 on both timepoints (PID 21; 1.27 ± 0.05 vs. 1.57±0.05; CIA+50 mg/kg NAR group vs. CIA+Vehicle group; *P* = 0.019) (Fig. 5A, B) (PID 35; 1.13 ± 0.06 vs. 1.41 ± 0.07; CIA+50 mg/kg NAR group vs. CIA+Vehicle group; *P* = 0.017) (Fig. 6A, B). In addition, the Western blot showed 50 mg/kg NAR significantly decreased the expression ratio of pCRMP2 S522 on both timepoints (PID 21; 0.62 ± 0.04 vs. 0.89 ± 0.04; CIA+50 mg/kg NAR group vs. CIA+Vehicle group; *P* = 0.016) (Fig. 5C, E, G) (PID 35; 0.70 ± 0.04 vs. 0.87 ± 0.04; CIA+50 mg/kg NAR group vs. CIA+Vehicle group; *P* = 0.015) (Fig. 6C, E, G). Since phosphorylation of CRMP2 at S522 is catalyzed by the kinase Cdk5, we next asked if Cdk5 itself could be dysregulated in CIA. On the day 28 (PID 21) and day 42 (PID 35) after initial immunization, the expression of Cdk5 were significantly increased in the CIA+Vehicle group (PID 21, 0.87 ± 0.03 vs. 0.54 ± 0.03; CIA+Vehicle group vs. Naive group; *P* < 0.01) (Fig. 5D) (PID 35, 0.95 ± 0.02 vs. 0.78 ± 0.04; CIA+Vehicle group vs. Naive group; *P* = 0.016) (Fig. 6D), while both elevations were significantly reverted by NAR (50 mg/kg) treatment (PID 21, 0.66 ± 0.03 vs. 0.87 ± 0.03; CIA+50 mg/kg NAR group vs. CIA+Vehicle group; *P* < 0.01; PID 35, 0.78±0.05 vs. 0.95 ± 0.02; CIA+50 mg/kg NAR group vs. CIA+Vehicle group; *P* = 0.016) (Figs. 5D and 6D). As a negative control, we tested a non-neuronal phosphorylation site of CRMP2, Y479, which was unchanged across all our treatment groups (PID 21, 0.85 ± 0.09 vs. 0.92 ± 0.09; CIA+50 mg/kg NAR group vs. CIA+Vehicle group; *P* = 0.98; PID 35, 0.61 ± 0.13 vs. 0.79 ± 0.11; CIA+50 mg/kg NAR group vs. CIA+Vehicle group; *P* = 0.67) (Figs. 5F and 6F). These results show that the analgesia and attenuation of spinal central sensitization by NAR might attribute to a reduction of the phosphorylation of neuronal CRMP2.

### Selective blocking phosphorylation of CRMP2 S522 causes attenuation of chronic pain in CIA model

**Fig. 7:**
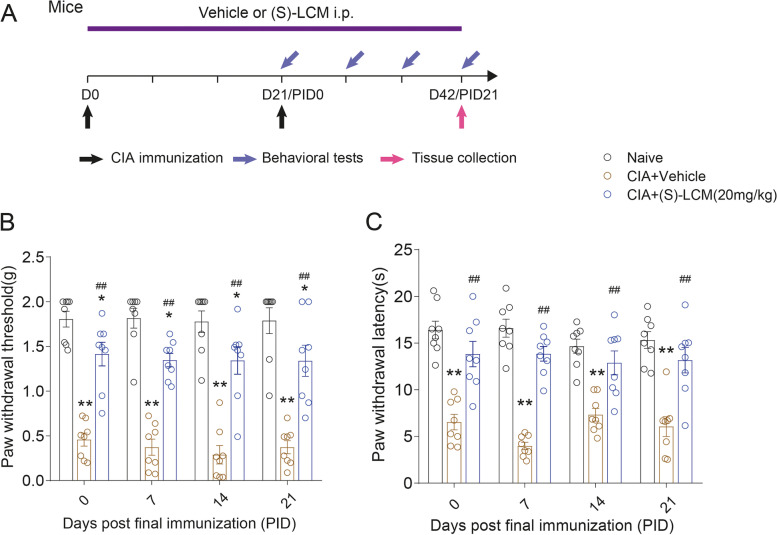
The CRMP2 S522 phosphorylation inhibitor (*S*)-LCM significantly reverses CIA-induced pain like behaviors. **A** Experimental design and treatment strategies. Paw withdrawal thresholds (**B**) and paw withdrawal latencies (**C**) of DBA1 mice were measured at the indicated days post initial day of immunization. (*S*)-LCM was given intraperitoneally at 20 mg/kg and behaviors were assessed 1 hour later. **P < 0.01 vs. naive group, ^##^*P* < 0.01 vs. CIA model group, by repeated-measures two-way ANOVA followed by post hoc Dunnett’s multiple comparisons test. The data are presented as means ± SEM (*n* = 8 per group). PID: Days post the second immunization

To further explore the contribution of neuronal CRMP2 pS522 phosphorylation in our model of CIA-induced arthritic pain, we used (*S*)-lacosamide as a selective inhibitor of CRMP2 phosphorylation by Cdk5 [38]. We recapitulated our model of CIA-induced arthritic pain in DBA/1 mice (sensitive to type II collagen and developed arthritis steadily and earlier) and treated the mice with (*S*)-lacosamide (20 mg/kg, i.p.) from the day of CIA initial immunization. On day 21 (PID 0) after the initial immunization, (*S*)-LCM significantly alleviated mechanical (1.41 ± 0.13g vs. 0.46 ± 0.07g, (*S*)-LCM group vs. CIA+Vehicle group, *P* < 0.01, *n* = 8) (Fig. 7B) and thermal pain sensitization (13.82 ± 1.35s vs. 6.54 ± 0.83s, (*S*)-LCM group vs. CIA+Vehicle group; *P* < 0.01, *n* = 8), which have lasted until day 42 (PID 21) (Fig. 7C). Thus, our data showed that CRMP2 phosphorylation by Cdk5 might be targeted pharmacology to reduce chronic pain in rheumatoid arthritis.

## Discussion

In this study, we demonstrated that naringenin administration significantly alleviated collagen-induced RA chronic pain, via (i) suppressing the spinal central sensitization, (ii) limiting spinal microgliosis and astrogliosis and (iii) attenuating the expression of phosphorylated spinal CRMP2. We further showed that CRMP2 phosphorylation by Cdk5 could be targeted pharmacologically with (*S*)-lacosamide to provide pain relief in rheumatoid arthritis. These findings highlight both naringenin and (*S*)-lacosamide as novel non-opioid therapeutics to treat pain and inflammation in arthritic pain.

RA patients are in need of efficient treatments to relieve their pain. NSAIDs can be used to improve pain in patients with RA, but as a single therapy, they have little benefit for patients for lack of effect on the central sensitization component of RA [39]. For tricyclic anti-depressants, randomized controlled trials for inflammatory arthritis (eight in nine trials for RA) found no evidence of short-term (1 week) benefits [40]. Other studies reported that opioids improved pain relief over placebo, but with an increased risk of adverse events and high likelihood of bias in patients [41]. Even though inflammation being optimally controlled, it is still indicative of a poor prognosis of RA patients with arthritic pain, and effective management of pain is therefore a common aspiration for both physicians and patients [19].

In this study, to investigate the appropriate dose of NAR application, we applied three drug concentrations of low (10 mg/kg), medium (20 mg/kg), and high (50 mg/kg), respectively. In addition, to investigate the effect of different concentrations of NAR in different time points of the disease, we added an additional time point, 28 days (PID 21) after initial immunization in the CIA model, which had the lowest values of mechanical and thermal pain thresholds in the animals of the CIA model group, to the observation node of the conventional experimental protocol. We found that NAR not only ameliorated the arthritis scores but also had a dose-dependent, long-lasting analgesic effect on collagen II-induced arthritic pain. No medication tolerance/desensitization of NAR-mediated pain-like behaviors relief were observed, and chronic administration of high doses of NAR did not result in any cytotoxicity throughout our study (data not shown). In addition, we found that NAR treatment provided an early alleviation (on PID 0) in heat hyperalgesia but had a delayed analgesic effect on the mechanical abnormal sensitivity test in RA animals, evidenced by the results that the NAR (20 mg/kg and 50 mg/kg) treatment almost reversed the decreased the paw withdraw latency to heat stimuli on day 7(PID 0) after the second (final) immunization (Fig. 1E). We infer that, in the early stages of arthritic pain progression, the abnormal pain threshold to heat stimuli might not get to the lowest level on D7 (PID 0). Furthermore, during chronic inflammation, the persistent upregulation of sodium channels (Nav1.8) in small fiber nerves participates in the sensitization of neurons to a noxious thermal stimulus [42]. We recently reported that NAR can induce analgesia by inhibiting the voltage-gated sodium channel NaV1.8 in DRG sensory neurons [29]. Thus, we speculated that the early analgesic effect on thermal hyperalgesia might be partially via the inhibition of C fiber NaV1.8, which was not excluded in our study.

cFos, as a member of immediate early genes, can express quickly and briefly when neurons respond to stimuli and is an indication of nociceptive stimulation and central sensitization. The expression level of cFos protein was higher in L4–L5 segments of the spinal lumbar vertebrae in animals after posterior traumatic sensory stimulation [43]. Additionally, glial cells are robustly activated in injured or diseased states and are involved in a variety of neuropathological changes, including chronic pain [44]. It was reported that early activation of joint nociceptors after injury triggers spinal microglial activation [45]. Microglia release cytokines (such as TNF-α and IL-18), growth factors (such as BDNF), and ATP, which in turn stimulates astrocytes and neurons to induce central sensitization [46]. We found that IBA-1 and GFAP, as well as cFos, were indeed elevated in the spinal dorsal horn in our CIA rat model. This could be curbed by NAR in a dose-dependent manner, although 50 mg/kg NAR marginally reduced cFos levels compared with CIA model group (*P* = 0.055) at 42 days (PID 35). Naringenin provides neuroprotection by inhibiting the pro-inflammatory pathway of activated BV2 microglia [47]. As autoimmune modeling was induced systematically, we did not compare the cFos expression between both sides. However, our study at least indicated that NAR might relieve chronic pain-like behaviors in a CIA rat model by inhibiting glial cell activation, as well as central sensitization.

CRMP2 is a member of the cytoplasmic phosphorylated protein CRMP1-5 family that plays an important role in chronic pain. We previously reported CRMP2 binds to and regulates CaV2.2 and NaV1.7 by maintaining their membrane localization and function [48]. After phosphorylation by CDK5 on its serine residue 522 (S522), CRMP2 potentiates the function of both Cav2.2 and Nav1.7 in DRG neurons which consequently mediates allodynic and hyperalgesic phenotypes in chronic pain [49]. Our previous studies additionally found that CRMP2 phosphorylated at S522 participates in spinal neurotransmission, reducing the distribution of spinal presynaptic CaV2.2 and NaV1.7 that involved in nociceptive signals, and plays an important role in the regulation of pathological pain [25]. Here, we explored if CRMP2 phosphorylation by Cdk5 could contribute to chronic pain in rheumatoid arthritis. We found that the phosphorylation of CRMP2 S522 was increased on day 28 (PID 21) and 42 (PID 35) after initial immunization and could be reduced using either NAR or (*S*)-lacosamide. NAR binding to CRMP2 was previously confirmed by using saturation transfer difference-nuclear magnetic resonance and microscale thermophoresis [29]. Besides, Lawal MF et al. also demonstrated the pharmacologic activity of NAR to selectively bind to CRMP2 and reduce its phosphorylation [30]. It might be a likely mechanism contributing to the analgesic effect on collagen-induced RA chronic pain of NAR in our study. Previously, CRMP2 function in the spinal dorsal horn was narrowed down to excitatory neuronal transmission. In agreement with this, we failed to detect any CRMP2 expression in spinal microglia and astrocytes, as well as in our recently published paper [24]. This indicated that, in addition to nerve injury, regulation of phosphorylation of CRMP2 in neurons might underlie central sensitization in rheumatoid arthritis too. To demonstrate this directly, we used the selective CRMP2 phosphorylation inhibitor (*S*)-lacosamide to rescue the sensitized mechanical and thermal thresholds in RA chronic pain. Taking all the evidence together, although it is still unclear whether NAR can directly block glial cell/astrocyte activation or it is downstream of reduced CRMP2 phosphorylation in dorsal horn neurons, we speculate that NAR may directly affect CRMP2 of neurons, regulate neuronal activity, and further suppressed central sensitization. Cross-talk between neuronal and glial mechanisms dominates the central sensitization. Microglia respond to several neuronal-derived signals (ATP, Fractalkine and MCP-1, etc.) which may lead to microglial activation [50]. Interestingly, the novel finding of an extracellular CRMP2 by Castillo and colleagues also raise the possibility of an extracellular CRMP2 participating in chronic pain under autoimmune disease, as well as the signaling between neuronal and glial mechanisms of central sensitization [51, 52].

While we focused on the neuronal component of sensory neuron sensitization via CRMP2 phosphorylation by Cdk5, CRMP2 could also play a role in the mitigation of autoimmune responses in RA. CRMP2 is expressed in lymphocytes and modulates their chemotaxis when phosphorylated by the kinase Tyr at Y479 [53]. To date, there are no available genetic or pharmacological tool to study the role of CRMP2 phosphorylation at Y479 in immune responses. Nevertheless, CRMP2 in immune cells might also be targeted to prevent or reverse the development of autoimmunity in rheumatoid arthritis; further investigations were needed.

## Limitations

Our study demonstrated that neuronal CRMP2 phosphorylation might be a promising target for reversal of spinal sensitization and arthritic pain improvement. However, what is the trigger that induces central sensitization under autoimmune disease is still unclear; more in vitro studies and even clinical trials are needed for further investigation.

## Conclusions

In summary, intervention of post-translational modification of spinal neuronal CRMP2 S522 to improve its mediation of pain is a promising way to develop non-opioid-dependent analgesia in RA chronic pain. Further efforts targeted on spinal neural CRMP2 post-translational modification may lead to novel drug discovery of analgesic compounds for intractable pain under autoimmune disease.

## Supplementary Information

**Additional file 1: Supplement Figure 1.** Collagen treatment successfully induced RA. A. ELISA was used to detect antibodies to type II collagen (A) or CCP (B). * *P* < 0.05 vs. naive group, by unpaired and two-tailed Student’s t-test (*n* = 5 per group).

**Additional file 2: Supplement Figure 2.** The CIA injected rats exhibited varying degrees of redness and swelling in their hind limbs. A. Naive rat hind limbs. B. CIA-induced rats hind limbs.

**Additional file 3: Supplement Figure 3.** Lack of co-localization between CRMP2 and IBA-1, CRMP2 and GFAP, and the co-localization between CRMP2 and NEUN. A. Representative micrographs showing expression of CRMP2 and IBA-1. B. Representative micrographs showing expression of CRMP2 and GFAP. All scale bars are 5 μm. C. Representative micrographs showing expression of CRMP2 and NEUN. All scale bars are 5 μm.

## Data Availability

The datasets used and/or analyzed during the current study are available from the corresponding author upon reasonable request.

## Abbreviations

CIA: Collagen-induced arthritis
RA: Rheumatoid arthritis
CNS: Central nervous system
(*S*)-LCM: (*S*)-lacosamide
DMARDs: Disease-modifying antirheumatic drugs
CRMPs: Collapsin response mediator proteins
NAR: Naringenin
