# Supplementary figures and images for: Neuronal CRMP2 phosphorylation inhibition by the flavonoid, naringenin, contributes to the reversal of spinal sensitization and arthritic pain improvement
Source: Arthritis Res Ther. 2022 Dec 23;24:277. doi: 10.1186/s13075-022-02975-8 (PMC9783725; doi:10.1186/s13075-022-02975-8)

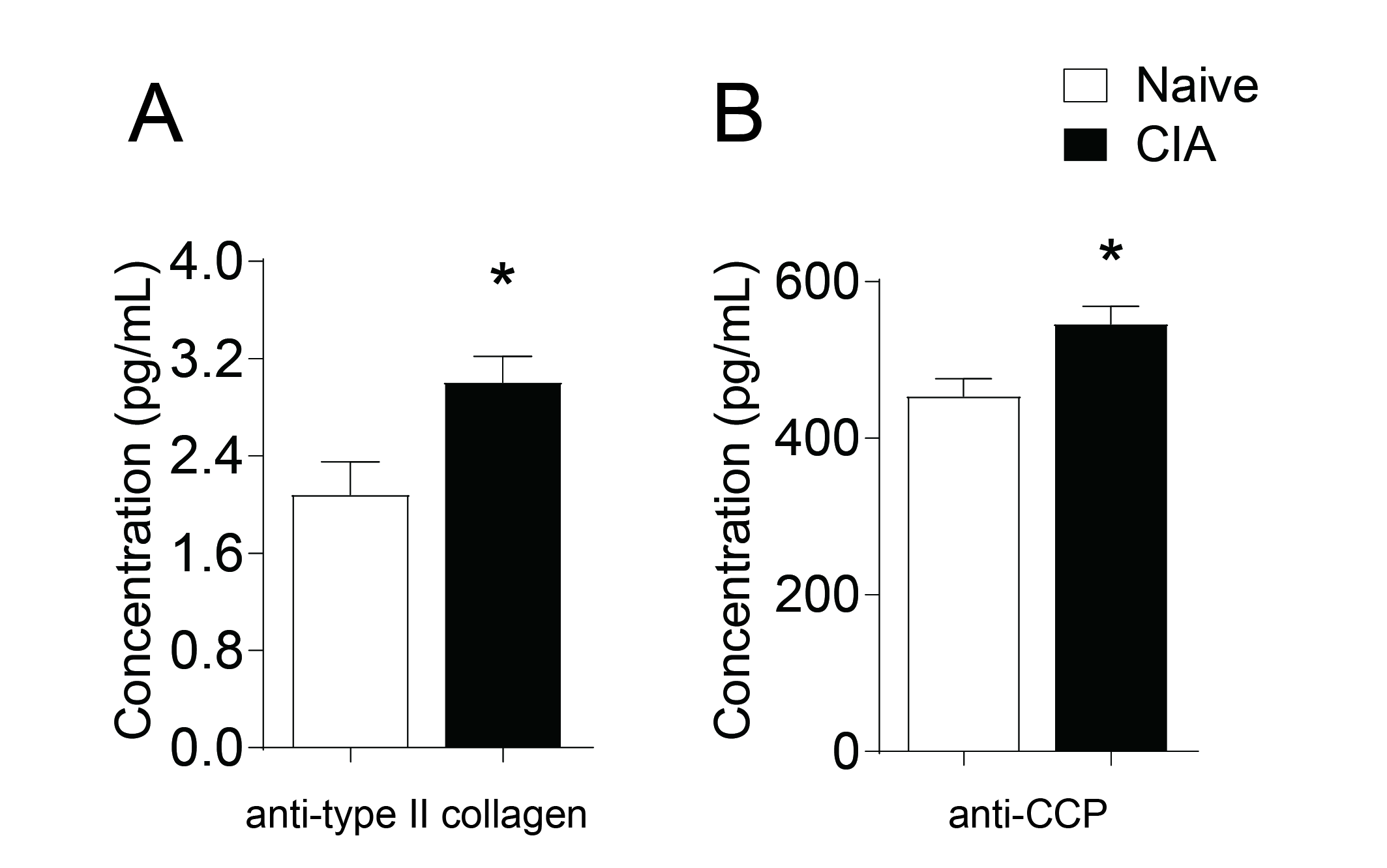

Supplement: Supplementary file 1 — Additional file 1: Supplement Figure 1. Collagen treatment successfully induced RA. A. ELISA was used to detect antibodies to type II collagen (A) or CCP (B). * P < 0.05 vs. naive group, by unpaired and two-tailed Student’s t-test (n = 5 per group). [file 13075_2022_2975_MOESM1_ESM.tif]

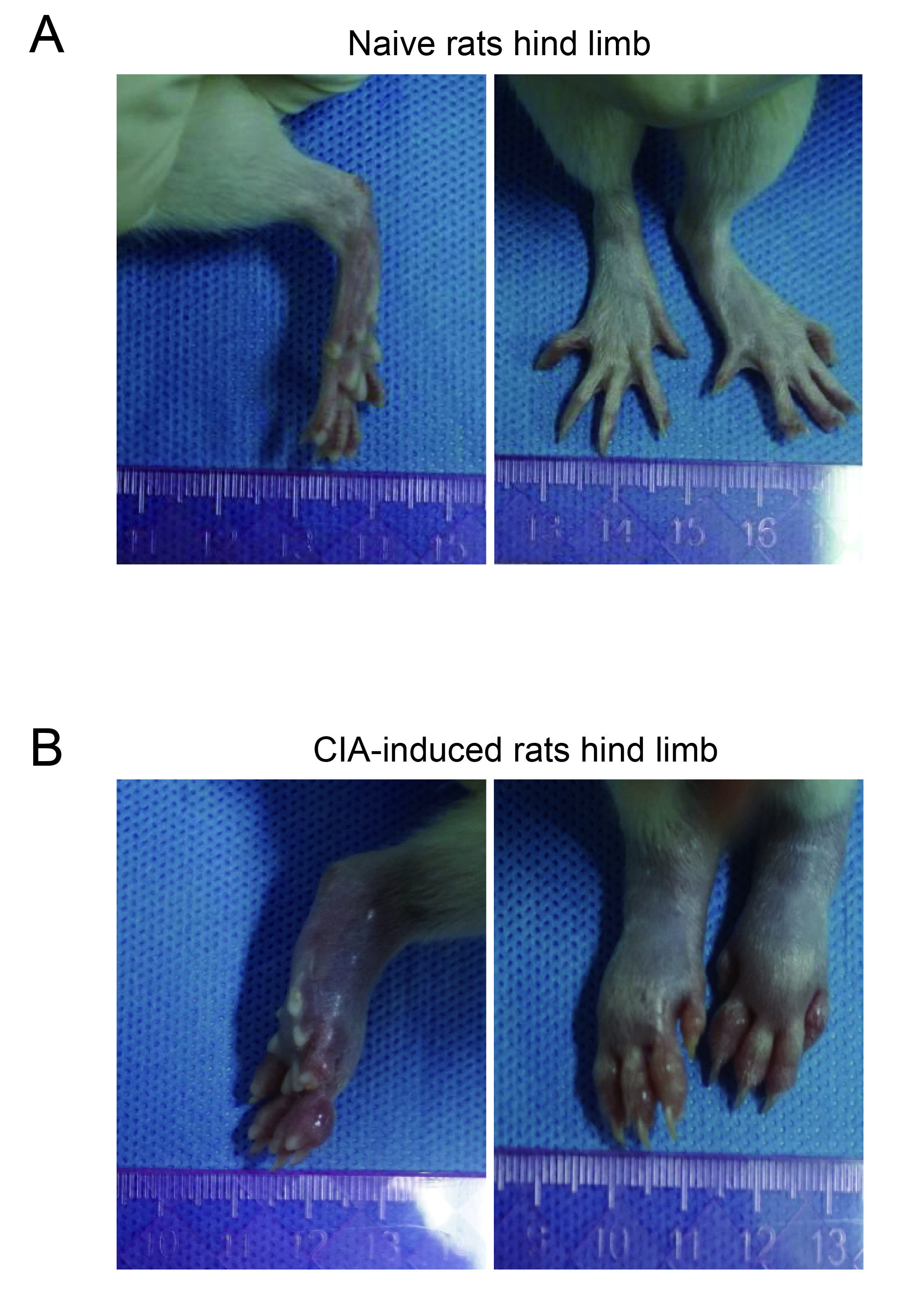

Supplement: Supplementary file 2 — Additional file 2: Supplement Figure 2. The CIA injected rats exhibited varying degrees of redness and swelling in their hind limbs. A. Naive rat hind limbs. B. CIA-induced rats hind limbs. [file 13075_2022_2975_MOESM2_ESM.tif]

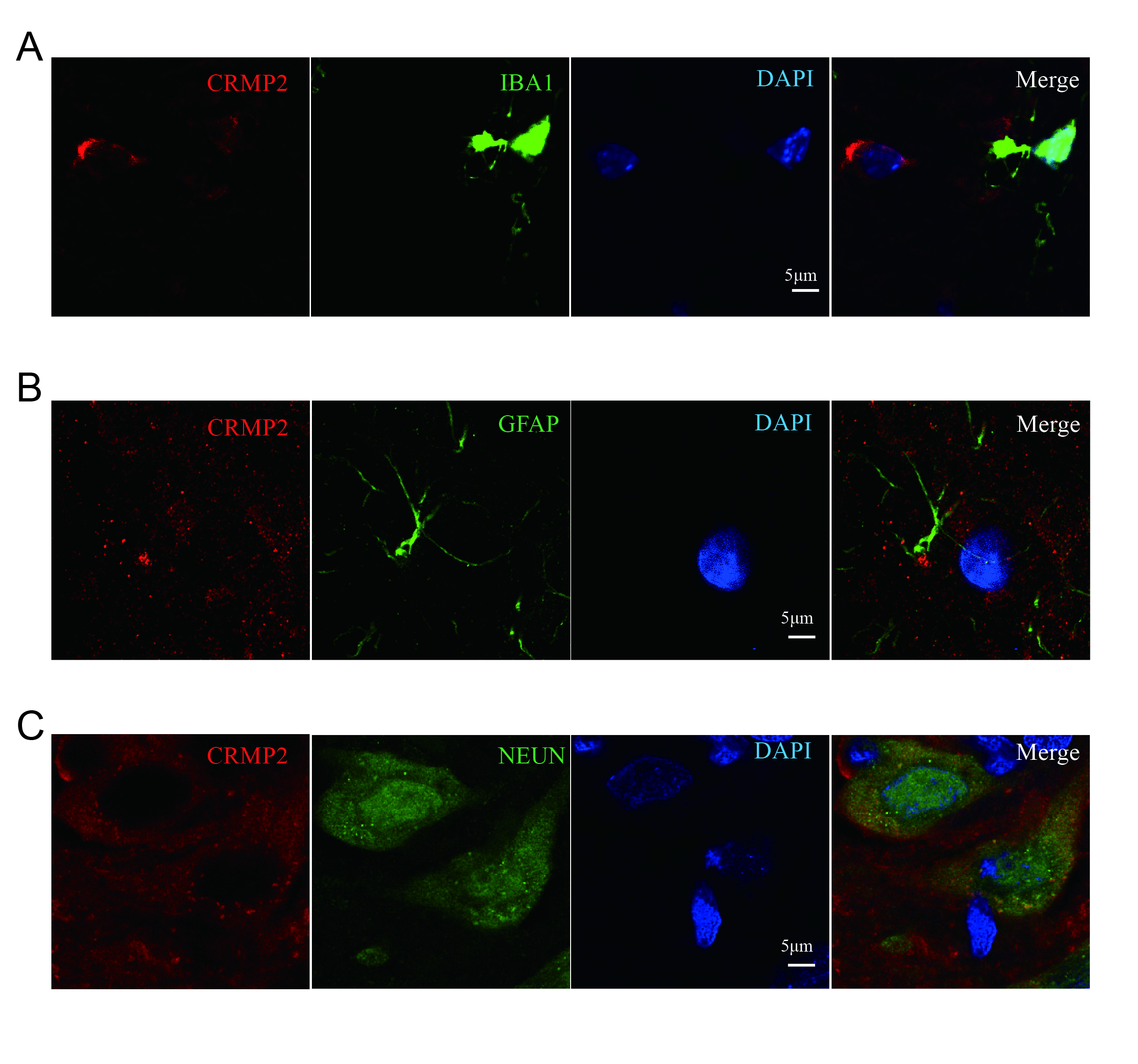

Supplement: Supplementary file 3 — Additional file 3: Supplement Figure 3. Lack of co-localization between CRMP2 and IBA-1, CRMP2 and GFAP, and the co-localization between CRMP2 and NEUN. A. Representative micrographs showing expression of CRMP2 and IBA-1. B. Representative micrographs showing expression of CRMP2 and GFAP. All scale bars are 5 μm. C. Representative micrographs showing expression of CRMP2 and NEUN. All scale bars are 5 μm. [file 13075_2022_2975_MOESM3_ESM.tif]
